# Photophysical Studies of Ruthenium-Based Complexes and the Performance of Nanostructured TiO_2_ Based Dye Sensitized Solar Cells

**DOI:** 10.35248/2157-7439.19.10.538

**Published:** 2019-12-07

**Authors:** Meser M Ali, Wasif Pervez, William Ghann, Jamal Uddin

**Affiliations:** 1 Department of Neurosurgery, Cellular and Molecular Imaging Laboratory, Henry Ford Hospital, Detroit, MI, USA; 2 Center for Nanotechnology, Department of Natural Sciences, Coppin State University, 2500 W. North Ave, Baltimore, MD, USA

**Keywords:** TiO_2_ nanoparticles, Solar cell, Ruthenium dye, DSSCs

## Abstract

Dye-sensitized solar cells (DSSCs) have attracted enormous attention in the last couple of decades due to their relatively small size, low cost and minimal environmental impact. DSSCs convert solar energy to electrical energy with the aid of a sensitizing dye. In this work, two ruthenium-based dyes, tris(bipyridine)ruthenium(II) chloride (Rubpy) and ruthenium(II)2,2’-bis(benzimidazol-2-yl)-4,4’-bipyridine (RubbbpyH_2_), were synthesized, characterized, and investigated for use as dye sensitizers in the fabrication of DSSCs. The photovoltaic performance of the ruthenium-based DSSCs was assessed. The solar-to-electric power efficiency of the RubbbpyH_2_ DSSC was 0.2% and that of the Rubpy was 0.03%. The RubbbpyH_2_ was also deprotonated and analyzed to study the effect of deprotonation on the efficiency of the solar cell. The deprotonated species, Rubbbpy, recorded an average efficiency of 0.12%. Thus, a change in pH did not enhance the efficiency of the solar cell. The cells were further characterized by impedance measurements. The photocurrent-photovoltage results were not consistent with the absorption spectra since Rubbbpy showed a more prominent band than RubbbpyH_2_ but had a lower efficiency.

## INTRODUCTION

Among the different types of renewable energy that have been investigated recently, solar power is one of the most efficient and inexhaustible sources available [1]. Although solar panels have grown in use, concerns have also grown regarding the various risks and disadvantages associated with traditional silicon-based solar panels, such as cost and the presence of toxic materials such as hexafluoroethane, lead, and polyvinyl fluoride. Dye-sensitized solar cells (DSSCs) have become attractive alternatives or complementary forms of solar cells owing to the fact that they are easily created, economical and fabricated with materials that are plentiful [2–4].

Work on DSSCs was first reported by O’Regan and Grätzel in 1991 [5]. Since then, there have been a plethora of publications dealing with the optimization of the various components of DSSCs with the goal of enhancing power conversion efficiency of the cells [6–12]. The DSSC is made up of three main parts: 1) a photoanode consisting of a sensitizing dye adsorbed on a semiconductor-coated fluorine doped tin oxide (FTO) glass; 2) an electrolyte made up of redox mediator; and 3) a counter electrode that facilitates redox reduction in the electrolyte.

In the presence of sunlight, the dye sensitizer absorbs photons leading to the ejection of an energized electron into the conduction band of the semiconductor. The injected electron is transported through the semiconductor to the conductive side of the FTO glass and subsequently through an external circuit to the counter electrode. At the counter electrode, the electron catalyzes the redox reaction in the electrolyte which in turn regenerates the oxidized dye. Thus, in DSSCs, charge separation occurs at the semiconductor-dye interfaces while the transport of charge is performed by the semiconductor and the electrolyte. This arrangement allows for optimization of the spectral properties to be carried out by modification of the dye alone. Similarly, the charge transport properties of the device could be enhanced by the optimization of the semiconductor and the electrolyte composition [13–15]. The many channels through which DSSCs can be optimized has given rise to a large number of papers reporting on studies carried out to improve the efficiency of the DSSC.

One particular area that has occupied the interest of most researchers is the dye sensitizers used in the solar cell. Both synthetic and natural dyes have been used with notable success. Natural dyes obtained from different parts of plants have been used in the fabrication of solar cells [16–21]. Although natural dyes are more environmentally friendly, low cost, and abundant in supply, their efficiencies are not as high as that of most synthetic dyes.

**Scheme 1:**
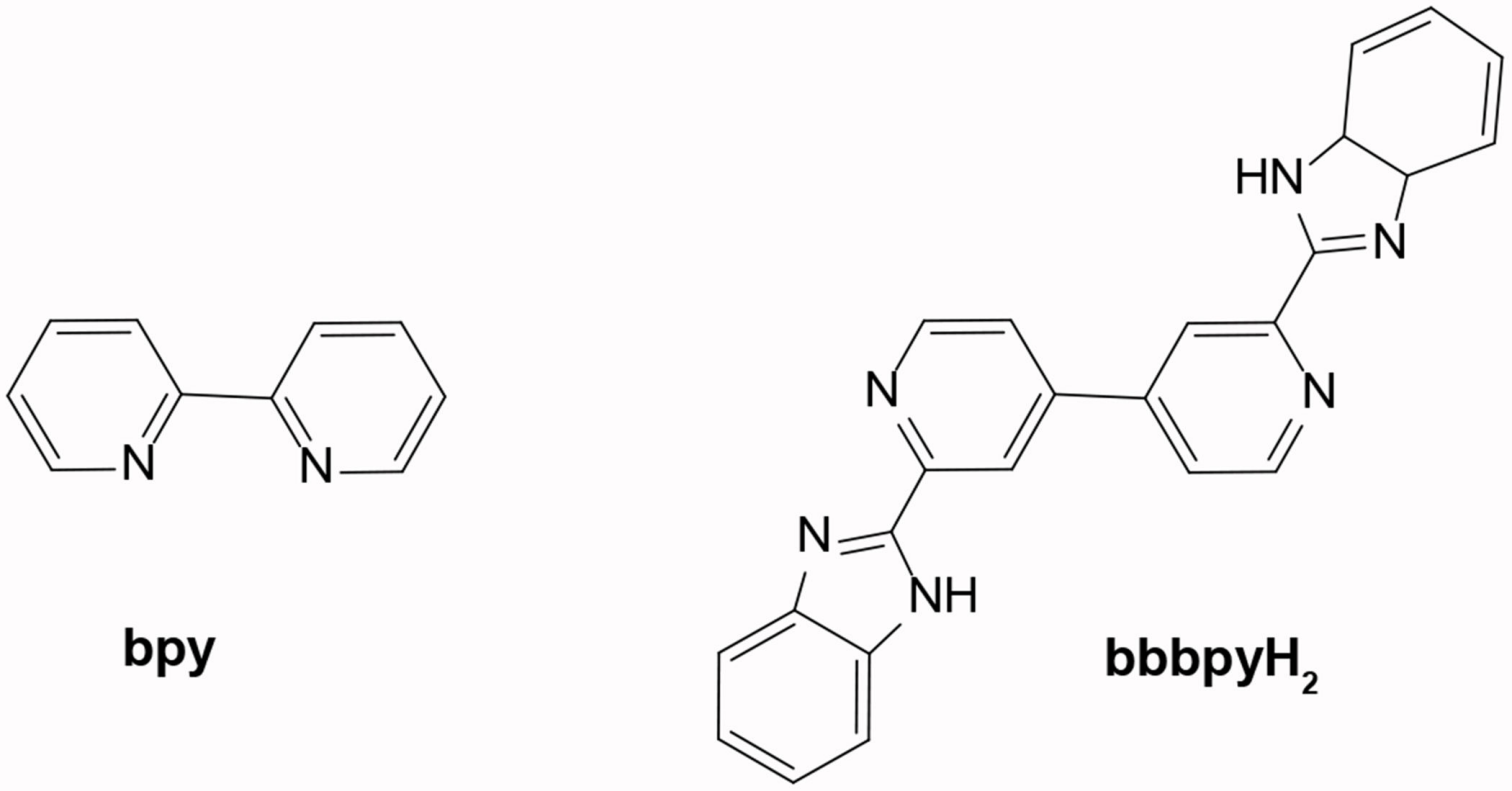
Schematic view of 2,2’-bipyridine (bpy) and bridging ligand 2,2′-bis(benzimidazol-2-yl)-4,4′-bipyridine (bbbpyH_2_).

Dyes that have been synthesized and applied in DSSCs include ruthenium complexes, [22,23] Porphyrin dyes, [24] and Cyanine dyes [25]. Among these groups, DSSCs using ruthenium complex dyes have consistently produced the best results due to their relatively long life in metal-to-ligand charge transfer (MLCT) properties. Most studies have reported using ruthenium trisbipyridin complexes as molecular devices for energy conversion. The modification or tuning of redox and photochemical properties have been reported by replacing bipyridin ligand with other nitrogen-containing heterocycles [26]. In addition, pH-dependent redox and photochemical properties have been reported in a ruthenium benzimidazole-based complex where imidazole moiety functions as a pH sensor [26]. In the present study, several ruthenium complexes containing bridging ligand 2,2′-bis(benzimidazol-2-yl)-4,4’-bipyridine (bbbpyH_2_) and 2,2’-bipyridine (Scheme 1) have been used as sensitizers for the fabrication of DSSCs.

The optical and structural properties of the dyes and the fabricated DSSCs were investigated. Also, the efficiencies of the fabricated solar cells were investigated via current and voltage measurement as well as by impedance spectroscopy, which examines the interface between the dye and TiO_2_.

## EXPERIMENTAL SECTION

### Materials

Titanium dioxide powder (Degussa P-25) was purchased from the Institute of Chemical Education, University of Wisconsin-Madison, Department of Chemistry, Madison, WI, USA. Fluorine tin oxide (FTO) coated glass slides were purchased from Hartford Glass Company, Hartford City, Indiana, USA. Sodium hydroxide (NaOH), ethanol (C_2_H_5_OH) and acetic acid (CH_3_CO_2_H) were purchased from Sigma-Aldrich (St. Louis, MS, USA) and were used without further purification.

### Characterization techniques

Absorption spectroscopy was carried out with UV-3600 Plus from Shimadzu, MD, USA. Emission spectroscopy was measured with RF-5301PC from Shimadzu, MD, USA. TiO_2_ paste was printed on FTO glass using WS-650 Series Spin Processor from Laurell Technologies Corporation, PA, USA. Carbon paint used in making cathode slides was purchased from Ted Pella, Inc., USA. The cell performance was measured using a 150 W fully reflective solar simulator with a standard illumination of air-mass 1.5 global (AM 1.5 G) having an irradiance of 100mW/cm^2^ (Sciencetech, Inc.) from London, Ontario, Canada. Reference 600 Potentiostat/Galvanostat/ZRA was purchased from GAMRY Instruments (Warminster, PA).

### Fabrication of DSSCs

The electrodes were prepared according to a previously published procedure with some modifications [27–30]. The working electrode was prepared by depositing a thin film of TiO_2_ on the conductive side of a fluorine doped tin oxide (FTO) glass slide using a spin coater and the film annealed at 380 °C for 2hours. The substrate was then immersed in dye for sensitization. The counter electrode (cathode) was prepared by printing colloidal graphite on FTO coated glass slide. The dye-sensitized slide and the carbon electrodes were assembled to form a solar cell by sandwiching a redox (I^−^/I_3_^−^) electrolyte solution.

### Synthesis of the bridging ligand 2,2′-bis(benzimidazol-2-yl)-4,4′-bipyridine (bbbpyH_2_)

The synthesis of bridging ligand 2,2′-bis(benzimidazol-2-yl)-4,4′-bipyridine (bbbpyH_2_) and its ruthenium complex was reported in details by Haga et al. [26].

## RESULT AND DISCUSSION

### UV Vis measurements

**Figure 1:**
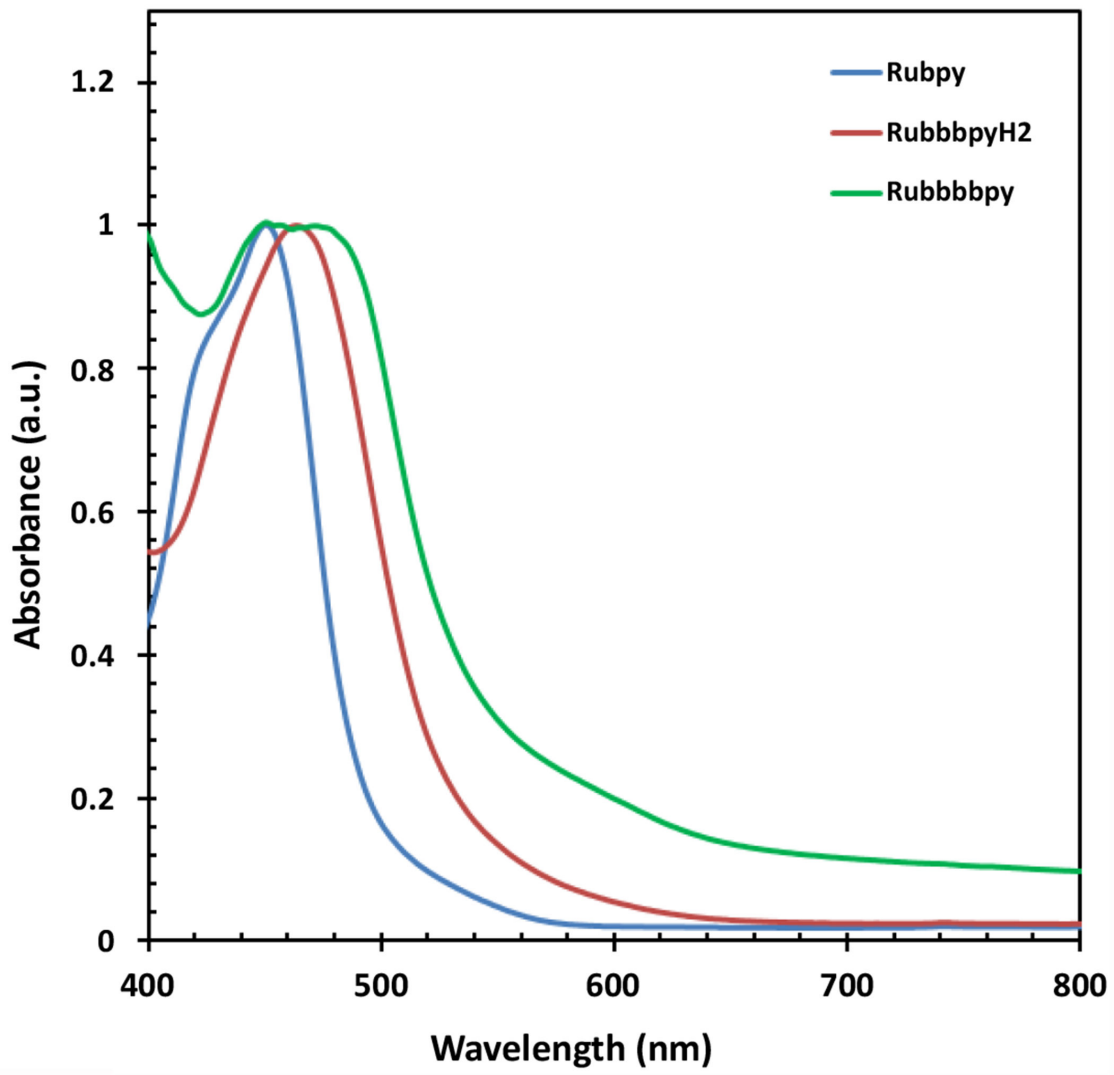
Absorption spectra of Rubpy, RubbbpyH_2_ and Rubbbpy in ethanol.

The UV-Vis measurement of the Rubpy, RubbbpyH_2_ and Rubbbpy (deprotonated form) were carried out to study the effect of absorption on the efficiency of the corresponding solar cell. The UV-vis spectrum of the Rubpy and RubbbpyH_2_ show single absorption peaks at 450 and 463 nm, respectively, as displayed in Figure 1. The UV-Vis of the Rubbbpy, however, is broad and exhibited dual peaks at 450 and 472 nm.

### Steady state fluorescence studies

**Figure 2:**
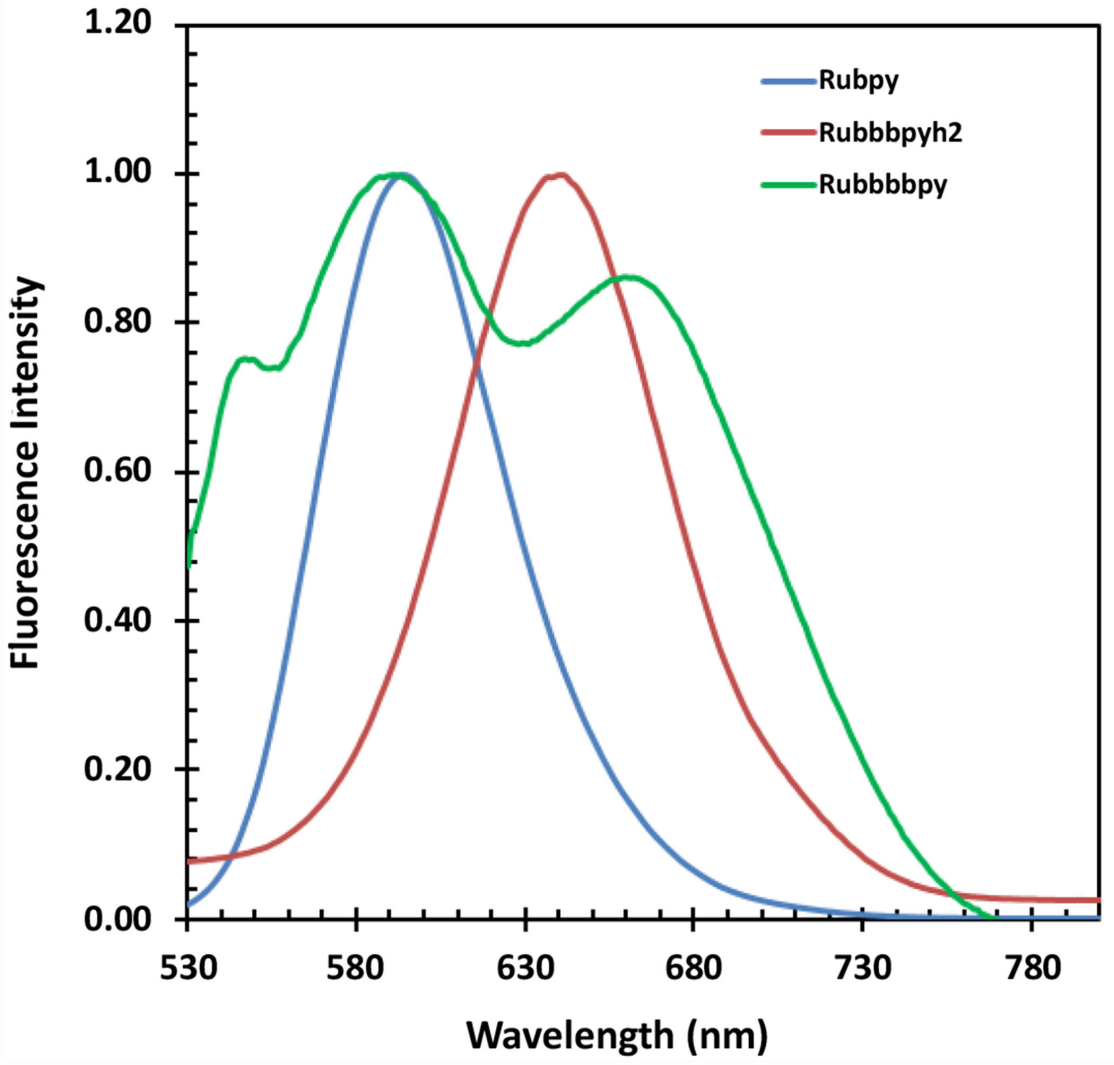
Emission spectra of Rubpy, RubbbpyH_2_ and Rubbbpy.

The steady state fluorescence spectra of the three dyes were taken as part of the photophysical studies on the dyes and are displayed in Figure 2. The measurements were carried out in ethanol with an exciting light of 600 nm. The emission maximum of Rubpy at 594 nm and that of the Rubbbpy is 641 nm but is red-shifted and broadened in the case of the deprotonated species.

### Fluorescence Lifetime measurement

**Figure 3:**
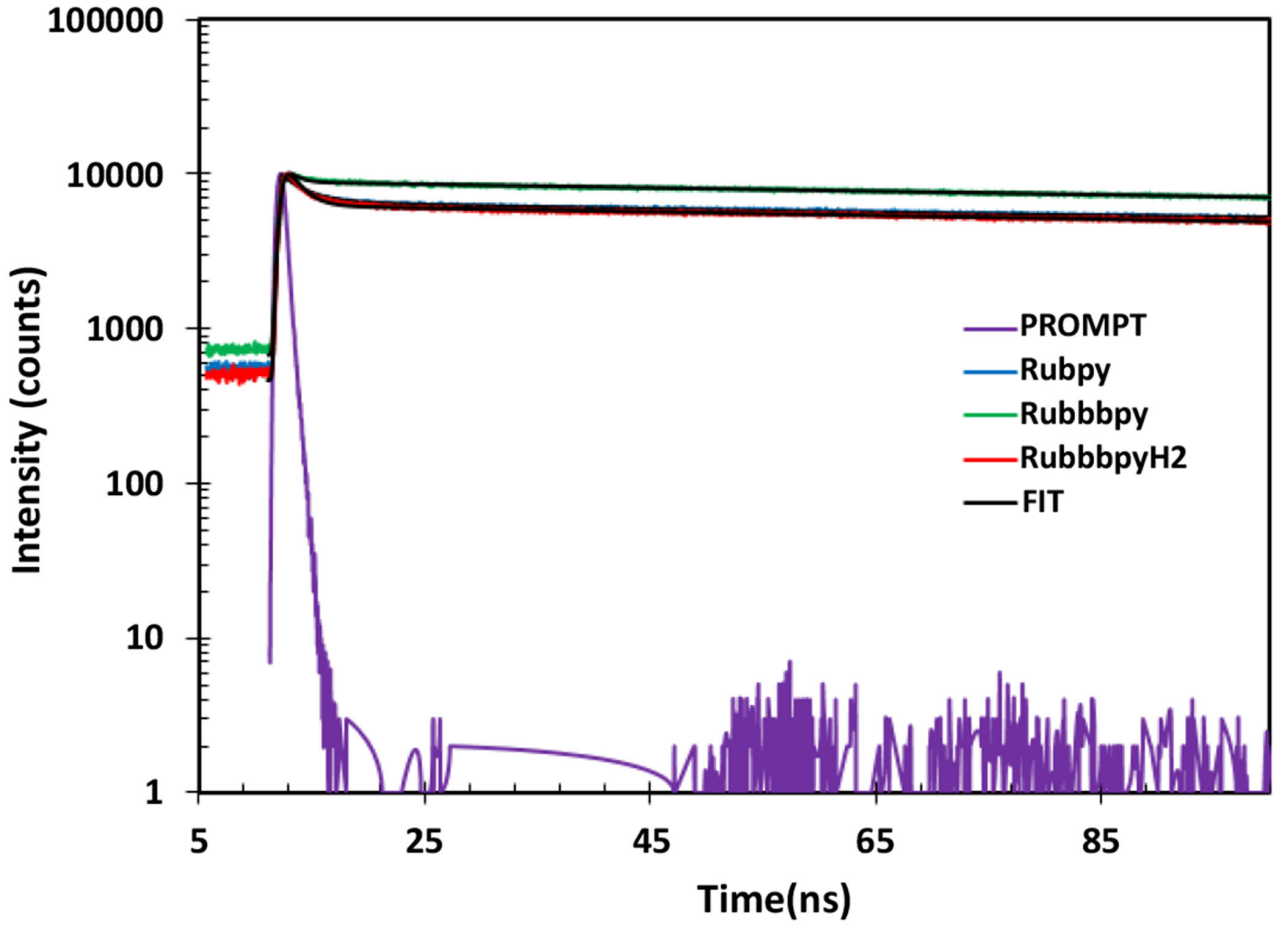
Lifetime measurements of Rubpy, RubbbpyH_2_ and Rubbbpy.

Fluorescence emission decay curves of the Rubpy, RubbbpyH_2_ and Rubbbpy were measured and the results are displayed in Figure 3 and Table 1. The measurements were conducted in ethanol. The results were fitted to the second exponential decay with a faster component of 22 ps, 13.4 ps, and 7.7 ps and a slower component of 347 ns, 332 ns, and 352 ns for the Rubpy, RubbbpyH_2_ and Rubbbpy, respectively. The values obtained are well within the lifetime values of 200-800 ns reported by Haga et al. [26] for mono- and dinuclear Ru complexes at room temperature. Dinuclear Ru complexes were specifically found to have a higher lifetime, however, water decreased the lifetime drastically. This decrease is attributed to the hydrogen-bonding interaction of the imino NH moiety of bbbpyH_2_ which acts as H^+^ donor and the water as H^+^ acceptor. Thus using ethanol as the solvent likely resulted in the lower lifetime due to the change in the excited-state properties.

### Fourier-transform infrared spectroscopy studies of dye-sensitized photoanode

**Figure 4:**
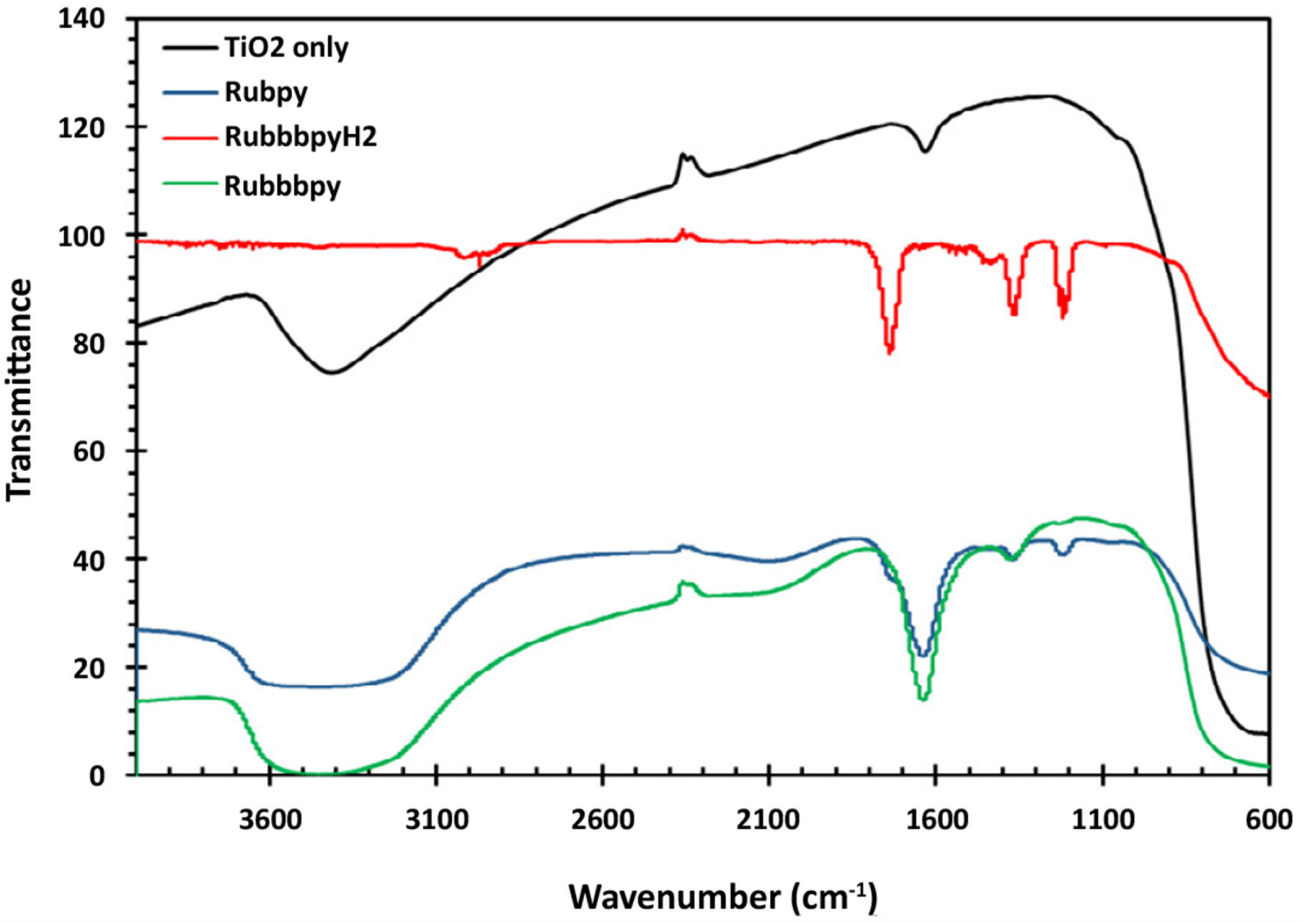
FTIR Spectra of the Rubpy, RubbbpyH_2_ and Rubbbpy sensitized TiO_2_ compared to that of blank TiO_2_.

Fourier-transform infrared spectroscopy (FTIR) studies were carried out to investigate the interaction of the TiO_2_ with different dyes that were utilized. The dyes usually need specific functional groups to effectively adsorb on the TiO_2_ film. In the presence of such functional groups, the dye adsorbs firmly to the titanium dioxide which results in the easy transport of charge and consequently a higher efficiency of the solar cell. The FTIR spectra of blank TiO_2_, Rubpy, RubbbpyH_2_ and Rubbbpy dye-sensitized titanium dioxide film are displayed in Figure 4. The peak at 3440 cm^−1^ is due to the presence of the hydroxyl group in the Rubbbpy.

### Raman studies of dye-sensitized photoanode

**Figure 5:**
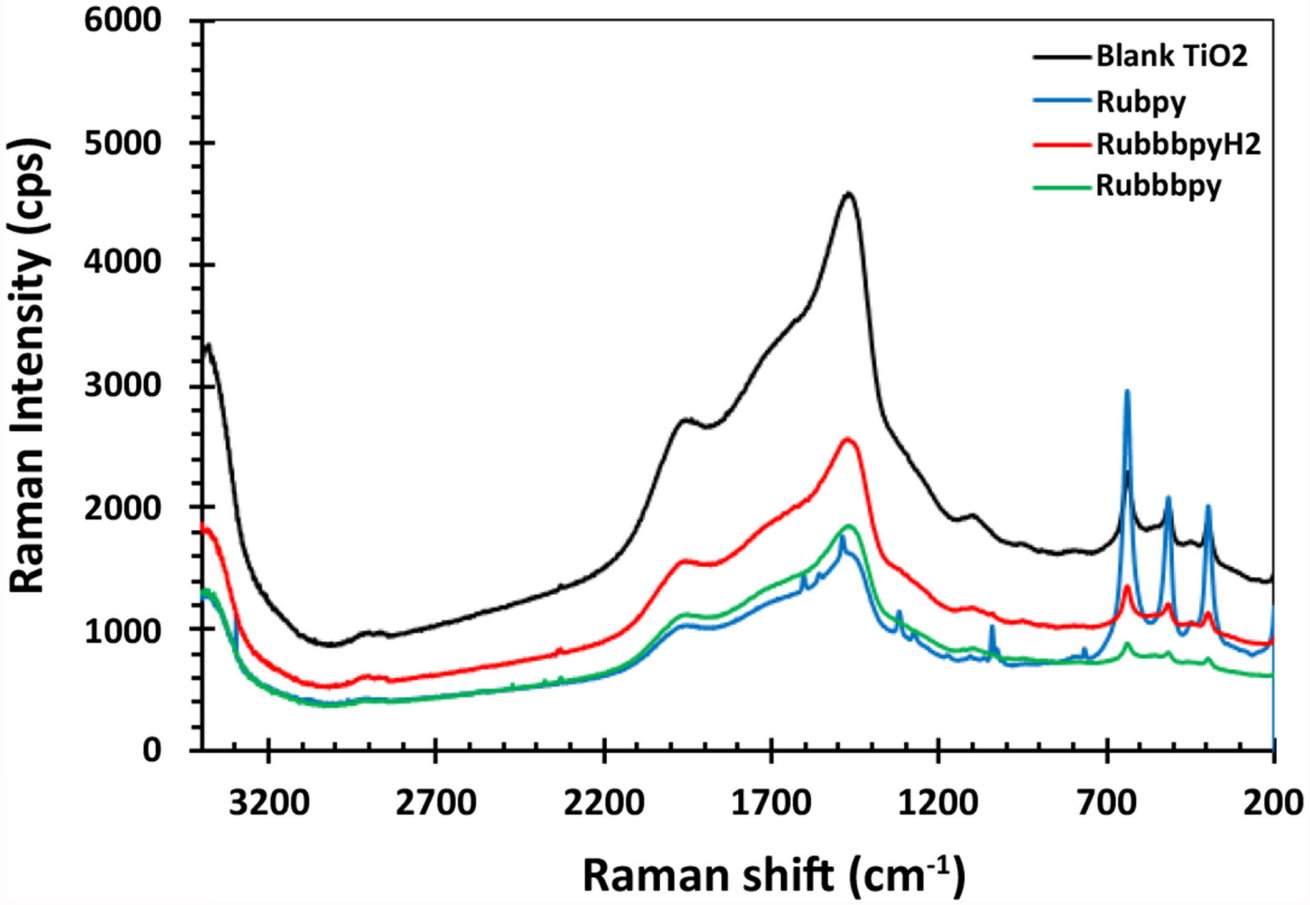
Raman Spectra of the Rubpy, RubbbpyH_2_ and Rubbbpy sensitized TiO_2_ compared to that of blank TiO_2_.

The Raman spectra of the dyes with titanium dioxide were measured to study the interaction of the dye with titanium dioxide. A comparison between Raman spectra of Rubpy dye, RubbbpyH_2_ and Rubbbpy with titanium dioxide was evaluated. Figure 5 shows spectra of the dyes adsorbed on the TiO_2_ surface, which is consistent with the Raman spectra of a typical ruthenium complex dye adsorbed to a titanium dioxide surface. The three bands at 395 cm^−1^, 513 cm^−1^, and 635 cm^−1^ are characteristic of the titanium dioxide. Two Raman spectra showing the characteristic bands at 1610 cm^−1^ and 1544 cm^−1^ are consistent with the ruthenium-based dyes.

### Current-voltage characteristics of Rubpy, Rubbbpy, and RubbbpyH_2_ DSSCs

**Figure 6:**
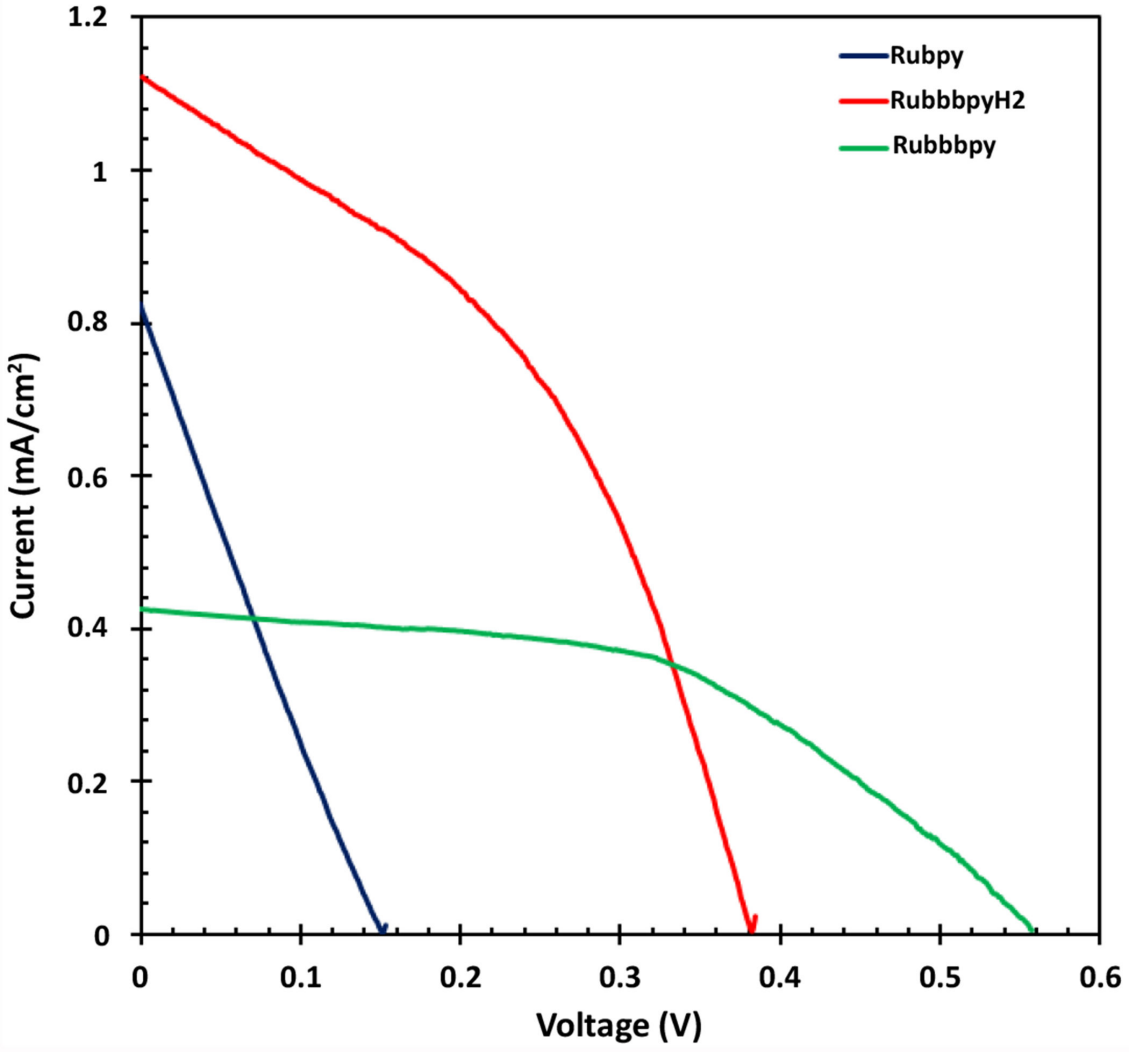
Photocurrent-voltage characteristics of Rubpy, Rubbbpy, and RubbbpyH_2_ dye- sensitized solar cells measured under illumination of 100 mW/cm^2^ (1.5 AM).

The current-voltage characteristics of the samples under simulated solar irradiation of AM 1.5 G were carried out to study the differences in the efficiency of solar cells. The results of the photocurrent-voltage measurements are displayed in Figure 6 and Table 2. The solar-to-electric power efficiency of the Rubpy DSSC was 0.029% whereas that of Rubbbpy, and RubbbpyH_2_ DSSCs was 0.118% and 0.201%, respectively. With deprotonation using sodium methoxide, the expectation was an improved interaction of the dye with titanium dioxide resulting in a higher efficiency. However, the efficiency of the RubbbpyH_2_ was found to be higher than that of the DSSCs of the deprotonated species. Thus, whereas the binding might have improved, it did not translate into a higher yield of the electric power.

### Electrochemical impedance spectroscopy

**Figure 7:**
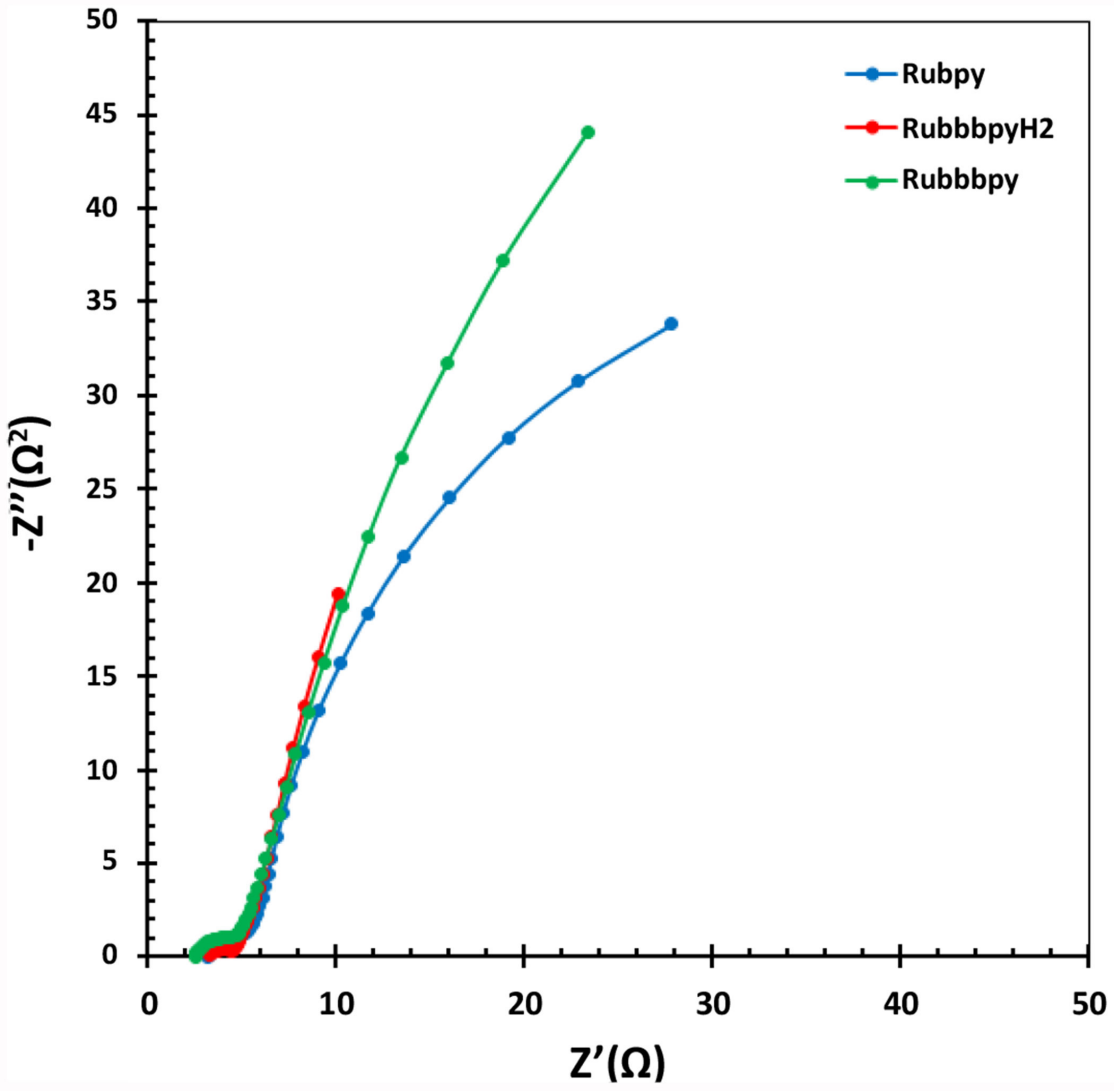
Nyquist plots for the fabricated Rubpy, Rubbbpy, and RubbbpyH_2_ dye sensitized solar cells showing differences in the resistance to charge transfer.

**Figure 8:**
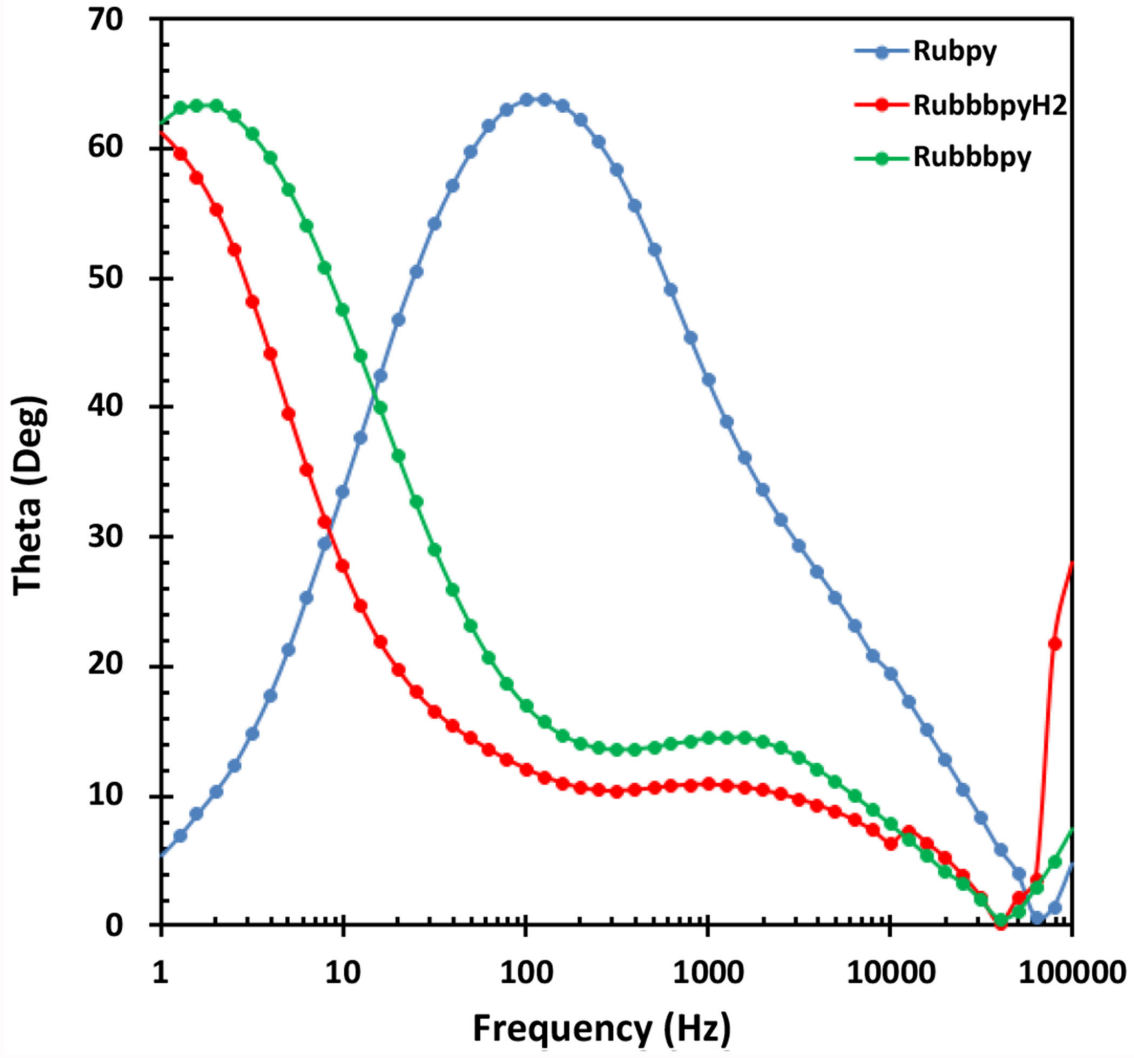
Bode plots for the fabricated Rubpy, Rubbbpy, and RubbbpyH_2_ dye sensitized solar cells.

Electrical impedance spectroscopy (EIS) is an important electrochemical technique normally used for investigation of charge carrier dynamics in DSSCs. EIS was employed in this work to characterize the three different DSSC samples. The results of the EIS measurement are presented in a Nyquist plot in Figure 7 and in a Bode plot in Figure 8. The measurements were undertaken in the frequency range of 0.01 Hz to 100 KHz. The two semicircles displayed in the Nyquist plot indicate the electron transfer processes between the dye-sensitized photoanode and the electrolyte interface.

## CONCLUSION

The photovoltaic performance of the RubpyCl_2_ DSSC was compared to that of RubbbpyH_2_ and its deprotonated form to study the effect of the imidazole group and pH on the efficiency of solar cells. The dyes were first characterized with UV-Vis, Emission Spectroscopy and Lifetime measurements. The solar cell efficiency of the Rubpy was 0.029% and that of the RubbbpyH_2_ was 0.20%. The efficiency of the Rubbbpy was 0.118%. Although the effect of the pH was evident in the photophysical studies, there was no significant change in the photovoltaic properties of the resulting DSSCs.

## Figures and Tables

**Table 1:** Fluorescence lifetime measurement of Rubpy, RubbbpyH_2_ and Rubbbpy.

Sample	Lifetime	Standard Deviation (*σ*)	Lifetime	Standard Deviation (*σ*)
	(*τ*_1_) (ps)		(*τ*_1_) (ns)	
Rubpy	22	2.47E-12	347	1.84E-09
RubbbpyH_2_	13.4	1.93E-11	332	1.44-9
Rubbbpy	7.7	2.1E-12s	352	2.70E-11

**Table 2:** Current voltage characteristics of Rubpy, RubbbpyH2 and Rubbbpy dye-sensitized solar cells.

	V_oc_	I_SC_ (mA/cm^2^)	V_mp_	I_mp_ (mA/cm^2^)	Fill Factor (%)	Efficiency (%)
	(V)		(V)			
Rubpy	0.15	0.83	0.07	0.41	0.23	0.03
RubbbpyH_2_	0.38	1.04	0.25	0.8	0.5	0.2
Rubbbpy	0.56	0.43	0.35	0.34	0.5	0.12
